# What's the problem? A response to "secular humanism and scientific psychiatry"

**DOI:** 10.1186/1747-5341-1-6

**Published:** 2006-04-25

**Authors:** Derek Bolton

**Affiliations:** 1 Institute of Psychiatry, Kings College London, London, UK

## Abstract

Notwithstanding the interest and importance of Szasz's position, it neglects the phenomena, the real problems which take people to the clinic seeking treatment, and the conditionality of the notion of individual responsibility.

The sociology of psychiatry – the ways in which psychiatry is shaped by and shapes wider society and its values – is a matter of enormous importance, because psychiatry has major impacts of many people's lives, and it costs a lot of money. Important but neglected, with some brave exceptions, among whom Szasz has ranked among the best few for decades. One writer, on the other hand, especially one who wants to make a point, tends to pursue one point, and it is up to the reader to consider other points of view, pros and cons, problems which are not addressed as well as those which are solved. This present piece by Szasz is a cogent and provocative summary of much of what he has long argued, in the form of a proposal that secular humanism is incompatible with scientific psychiatry.

Szasz sketches the two players, citing the website of the Council for Secular Humanism as follows:

"Secular humanists reject authoritarian beliefs. They affirm that we must take responsibility for our own lives and the communities and world in which we live. Secular humanism emphasizes reason and scientific inquiry, individual freedom and responsibility, human values and compassion, and the need for tolerance and cooperation."

And then psychiatry is characterised like this:

The term "psychiatry" refers to both the principles and practices of this ostensibly medical specialty. It is necessary to emphasize at the outset that, unlike typical medical practices based on consent, typical psychiatric practices rest on coercion.'

I imagine the *credo *of secular humanism would attract a lot of well-wishers, at face value, give or take some interpretations in tricky cases of some of the key terms especially in the second quoted sentence; but anyway, generally fine sentiments. Psychiatry on the other hand gets off to terrible start, what with having with an ill-defined basis, medical but not really, and being typically coercive. Since secular humanism values freedom, among other things, and psychiatry operates by coercion, the answer to the question Szasz raises at the start whether they are compatible is just 'no'. The problem solves itself as soon as it is formulated, and the rest of the piece is an elaboration of the point.

Just the definitions of secular humanism on the one hand and psychiatry on the other shows up the strength of Szasz's conclusion clearly, but the easiness of it all signifies that hard problems are left invisible. What are the hard problems?

First, much of mental health services are provided to voluntary consumers who walk through the clinic door asking for treatment. What can the kind of position adopted by Szasz say about this? Perhaps that these people are victims of a delusion, of the same myth that defines psychiatry? Or that they wish to shirk responsibility for their feelings and behaviour? Deluded or feckless they may be, but in any case apparently not responsibly seeking help for something reasonably called a 'mental health problem'. So for example, consider the case of a 17 year old young man finding that he is continually having thoughts that his parents may have an accident, that to stop this happening he is convinced that he has to undo the thoughts by repeating what he was doing when he has them without having them, that this takes hours, gets in the way of revising for his state exams, seeing friends and sleeping, believes that this all makes no real sense, finds out on the net that he has what is called OCD and that it is treatable (stoppable) by CBT, so goes looking for it. Is such a man deluded, feckless, or responsibly taking care of himself? Do we have to choose one of the first two options? Or can we go with the third? If the first two, what should the young man be doing in his predicament? Or is it, despite appearances, not a predicament at all? What should the therapist he contacts do? What does the position adopted by Szasz recommend we say and do in such a case? These are the day to day problems and questions that are apparently passed by in Szasz's critique of psychiatry. And abstract debates about such as individual freedom, and whether so-called mental health problems are or are not brain disorders, just get us to ignore them.

In the coercive case, what should be done instead? What should be done with a person – let us say a mother of two young children and a new baby to make the case a hard one – who has so-called 'post-natal depression' and in this so-called depressed state expresses clear intent to kill herself, with attempts, and a clear choice not to have treatment? What should be done? Leave her to it?

These are the practical problems, in both kinds of case, voluntary treatment-seeking and coercive treatment, that keep people with the problems, their families, and the professionals, awake at night. We all need to know what we should do instead, if not follow the current arrangements. The *credo *of the Council for Secular Humanism was presumably not designed to get involved with these practical problems, and nor is Szasz's piece.

Behind all this it may be that the real issue is not about what are called mental health problems and its management, which would be why little is said about them, but about the role of the state. After introducing the definitions that make it clear that psychiatry is incompatible with the ideals of secular humanism, Szasz continues:

In a free society, most social relations between adults are consensual. Consensual relations – in business, medicine, religion, and psychiatry – pose no special legal or political problems. In contrast, coercive relations – one person authorized to use the power of the state to compel another person to do or abstain from an action of his choice – are inherently political and morally problematic.

It is a secure and comfortable world in which consensual relations pose no special legal or political problems, in which these problems are raised only by a coercive state: one source of problems, which comes with its own built-in solution, curtailing the power of the state. But what here is said about consensual relations between two parties that spike a third – not an unusual position after all? Or are these consensual relations that damage a third party illegal, regulated by law, backed up by a coercive state, which would then not be so bad after all? How is the argument meant to run here? Apparently the state has to regulate the behaviour of citizens and corporations in many ways, to make it work at all. There is after all no property without property law, no trade without contracts, nor indeed is there freedom to act without law which protects the individual against coercion by another.

Further, law itself rests on the notion of individual responsibility for action. This notion is fundamental to the kind of position adopted by Szasz, fundamental and unquestioned, secure and comfortable. But the assumption of individual responsibility only seems this way when we ignore all the many kinds of problematic cases in which it is questionable, problems that exercise the legislators and the courts, how to judge individual responsibility in cases involving such as corporate misdemeanours or war crimes, for example, or involving minors, the seriously physically ill, the state of people in altered/uncharacteristic states of consciousness for various reasons and causes, and of people with severe so-called 'mental health problems'. If it is to be understood adequately, mental health law that warrants coercion has to be discussed in the context of problems such as these that interrogate the assumption of individual responsibility and freedom, not only in the assumption of free, autonomous, responsible, value-laden and cooperative individuals managing things for themselves for the best.

